# IsoFoodTrack: a comprehensive database and management system based on stable isotope ratio analysis for combating food fraud

**DOI:** 10.3389/fnut.2025.1516521

**Published:** 2025-02-19

**Authors:** Cathrine Terro, Robert Modic, Matevž Ogrinc, Andraž Simčič, Jan Drole, Tome Eftimov, Barbara Koroušić Seljak, Nives Ogrinc

**Affiliations:** ^1^Department of Environmental Sciences, Jožef Stefan Institute, Ljubljana, Slovenia; ^2^Jožef Stefan International Postgraduate School, Ljubljana, Slovenia; ^3^Computer Systems, Jožef Stefan Institute, Ljubljana, Slovenia

**Keywords:** database, stable isotope ratio analysis, elemental composition, food fraud, authenticity, interoperability

## Abstract

The IsoFoodTrack database is a comprehensive, scalable, and flexible platform designed to manage isotopic and elemental composition data for a wide range of food commodities. It supports research in food authenticity and fraud detection by integrating isotopic data with rich metadata, including geographical, production, and methodological details. The database is built for scalability, allowing the addition of new commodities, analytical methods, and metadata fields, while ensuring interoperability with external databases through standardized formats and API integration. Based on the data collected in IsoFoodTrack using statistical, chemometric and machine learning approaches it has a capability to identify and classify the origin of food commodities. IsoFoodTrack also supports isotope mapping (isoscapes), providing spatially continuous predictions that enhance the detection of food fraud. Rigorous quality control measures ensure high data reliability, and the user-friendly web interface facilitates easy access and visualization. Openly accessible through platforms like National Center for Biomedical Ontology (NCBO) BioPortal, IsoFoodTrack is positioned for future expansion and integration of open-access data, making it a vital tool for researchers and regulatory agencies in ensuring food authenticity and traceability.

## 1 Introduction

Food fraud, which refers to the economically motivated adulteration and mislabeling of food products, continues to be a major issue for food producers as well as consumers. Among the techniques available for detecting fraud, stable isotope fingerprinting is leading the way in establishing the authenticity and geographical origin of food products. This choice is based on the fact that the distribution of stable isotopes of carbon (^12^C, ^13^C), nitrogen (^14^N, ^15^N), sulfur (^32^S, ^34^S), hydrogen (^1^H, ^2^H), and oxygen (^16^O, ^18^O)^1^ is influenced by fractionation processes linked to local climate, geology, and soil characteristics (1, 2). These processes result in varying rates of isotope transfer from natural sources such as water, soil, and the atmosphere to plant or animal tissues. For example, the isotope ratios in water (^2^H/^1^H and ^18^O/^16^O) provides critical information about local precipitation, surface water, and groundwater, influenced by factors like latitude, altitude, distance from the sea, precipitation levels, and evapotranspiration. The verification of regional origin becomes even more robust when isotope data are combined with elemental composition profiles (3). However, to determine authenticity, a suitable reference dataset of analyzed authentic products is required. This dataset should include samples representative of a wide range of geographical, seasonal, dietary, and production conditions. Authenticity is then assessed by comparing the values found in commercial samples with the limits estimated from the reference dataset, using a suitable statistical model to evaluate the best fit. These databases also need to be continuously curated and kept up to date, which is a considerable task, given the amount of variation that needs to be included.

A prime example of a well-established database is the European Wine DataBank, which the European Commission has maintained for over 20 years (4). However, while some databases are publicly available, many others are not freely shared due to intellectual property concerns and differences in sample pre-treatment methods. Nevertheless, the use of isotope databases is expanding; for example, the Stable Isotope Ratio Analysis (SIRA) database is already being applied to products like Parma ham, Grana Padano cheese, and Parmigiano Reggiano in Italy (5). Other examples include the pork origin database managed by the UK’s Agriculture and Horticulture Development Board (AHDB), the egg database Kontrollierte Alternative Tierhaltungsformen (KAT), and asparagus databases in German food control labs.

To address the gaps in the availability and accessibility of such data, we have developed a comprehensive database and database management system (DBMS) called IsoFoodTrack.^2^ This system provides extensive data on the stable isotopes of light elements and the elemental composition of authentic samples from various food commodities such as oils, milk and dairy products, meat, spices, truffles, seafood and vegetables. Furthermore, IsoFoodTrack is designed to be interoperable, allowing connection with other databases or centralized repositories. IsoFoodTrack represents a significant advancement over traditional food databases by prioritizing both accessibility and standardization. It incorporates open-access principles, ensuring that researchers from diverse regions can utilize its resources without significant barriers. Additionally, the database integrates standardized metadata protocols and harmonized data entry formats, which streamline cross-study comparisons and enhance reproducibility.

Additionally, there is a growing movement toward creating a centralized repository for isotopic data, as proposed by Pauli et al. (6), with the development of IsoBank. IsoBank aims to function similarly to GenBank in the field of genetics, serving as both an aggregator and repository of open-access isotope data. IsoBank is designed as a general-purpose repository for stable isotope data across all disciplines. It supports the storage and retrieval of isotope measurements irrespective of their context. It serves a broad research community, including fields like ecology, geology, archeology, and biology, among others. This resource will promote interdisciplinary research, facilitate data-sharing, and provide valuable educational opportunities by offering real-world isotopic data for students and researchers alike. On the other hand, IsoFoodTrack database serves as a specialized database aimed at practical applications in food traceability and authenticity verification, prioritizing functionality tailored to its specific use case. Its scope is narrower, targeting applications in food science, agriculture, and regulatory frameworks.

In this paper, we present the IsoFoodTrack database as the first effort to organize open-access stable isotope data for food research. It is organized in different sections including: database design, methods and technical aspects. Section 5 details the validation of IsoFoodTrack, demonstrating its practical application, while section 6 deals with database curation and availability. Finally, section 7 concludes the paper by discussing key achievements and contributions.

## 2 Database design

The design of the IsoFoodTrack database is crucial for ensuring the effective management of isotopic and elemental composition data for various food commodities. The database was developed with a focus on scalability, flexibility, and data integrity, enabling researchers to store, retrieve, and analyze stable isotope data in a structured and efficient manner. This section outlines the key aspects of the database design, including the data model, schema design, relationships between entities, and considerations for maintaining accuracy and performance.

### 2.1 Data model

The IsoFoodTrack database follows a relational database model, which is well-suited for managing structured data with clearly defined relationships between entities. The relational model enables efficient querying of data, as well as maintaining consistency and data integrity through the use of primary and foreign key constraints. This design ensures that all data points, including isotopic ratios, elemental compositions, and metadata about samples, are properly linked to their relevant entities.

**Figure 1:**
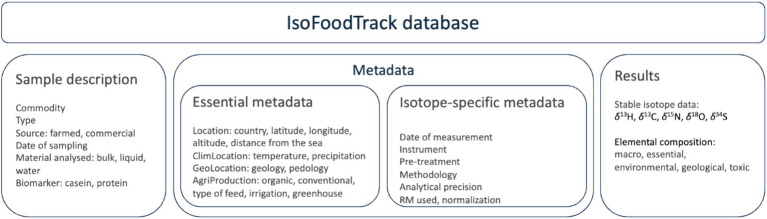
Structure of the IsoFoodTrack database.

The structure of IsoFoodTrack is presented in Figure 1.

The main entities in the database include:

- *Samples*: representing the physical food samples analyzed for isotope and elemental composition. This also includes the type of sample, source of sample (authentic, commercial), date of sampling and compound analysed: bulk sample (freeze-dry or liquid), sample water, extracted components, fatty acid.
- *Metadata*: the metadata cover two categories: (i) essential metadata, describing every data record, and (ii) isotope-specific metadata. The essential metadata includes *geographical data:* storing information about the geographical location of each sample, including details such as latitude, longitude, altitude, distance from the sea. In addition, the data on yearly average amount of precipitation, average temperature of the location, geology and pedology are also included.
  *Production data:* capturing details about the production and processing of the food samples, such as year of production, type of material (authentic, commercial), farming practices, seasonal information, and production methods (e.g., organic or conventional; if known).
  The *isotope-specific metadata* includes reference materials used to normalize the data. Stable isotope data are produced in a wide range of research and commercial laboratories. Although the methods by which the majority of data, i.e., bulk carbon (*δ*^13^C) and nitrogen (*δ*^15^N) stable isotope values, are standardized, laboratories often use slightly different protocols and different laboratory reference materials to normalize data to internationally accepted scales. Other isotopes (e.g., *δ*^2^H and *δ*^18^O) have more fundamental issues associated with comparability of measurements (7). Hydrogen in an exchangeable position (e.g., when bound to oxygen in a hydroxyl group, as in proteinaceous material) will exchange with atmospheric water vapor, leading to potentially erroneous results unless controlled. Thus, to ensure data robustness, quality and user confidence, all pertinent analytical information for each piece of data is recorded. IsoFoodTrack metadata includes sample pretreatment methods (e.g., lipid extraction), reference materials used, type of normalization (one, two, multi-point normalization) and instrumentation used.
- *Isotope data*: storing detailed information about the isotopic composition of each sample, including ratios of isotopes such as *δ*^2^H, *δ*^13^C, *δ*^15^N, *δ*^18^O and *δ*^34^S (in ‰).
- *Elemental data*: capturing elemental concentrations for key elements: B, Na, Mg, Al, P, S, K, Ca, V, Cr, Mn, Fe, Co, Cu, Zn, As, Se, Rb, Sr., Mo, Cd, Cs, Ba, Hg, Pb.

### 2.2 Scalability and interoperability

The design of IsoFoodTrack anticipates the continuous expansion of the database as new samples are collected and analyzed. As such, the database architecture is scalable, with the ability to accommodate additional tables for new food commodities or analytical methods such as compound specific analysis.

## 3 Methods

The development of the IsoFoodTrack database involved several key stages, including the collection and preparation of authentic food samples, the analysis of isotopic and elemental compositions, the organization and management of data, and the validation of the database for practical applications in food authenticity testing. This section describes the methodology employed to create and curate the IsoFoodTrack database.

### 3.1 Sample collection and preparation

Sample collection represents a crucial step in the formation of the IsoFoodTrack database, and in order to ensure the accuracy of a food authenticity database, authentic samples must be used. Ideally, samples should be collected from primary producers by impartial collectors to ensure traceability and authenticity. In comparison, retail samples are less reliable due to possible contamination in the supply chain. When creating the database, sample size and variety are also important and should reflect natural variations, e.g., geography, breed, and climate. Additionally, the database should be validated for its intended use, and statistical analysis should be considered when determining sample size. The selection of reference data from the database is crucial and should be left to experts only (8, 9).

In our case, the sampling follows an appropriate protocol developed for various applications to mitigate potential biases caused by the overrepresentation of specific regions. This protocol is based on our prior experience and expert knowledge. To ensure a robust and representative dataset, samples were collected using the following criteria:

*Geographical diversity*: samples were sourced from a wide range of geographical locations, including different latitudes, altitudes, and climatic zones. This ensures that the database captures the natural variation in isotopic and elemental signatures that arise from environmental factors such as local precipitation, soil types, and temperature. In case of Slovenia, to account for natural variability, sampling was designed to reflect Slovenia’s regionalization, which is divided into four distinct geographical regions: Dinaric, Mediterranean, Alpine, and Pannonian.

*Seasonal and temporal variation*: samples were collected over multiple growing seasons and harvest periods to account for seasonal changes in isotopic and elemental composition. These variations can be influenced by factors such as precipitation levels and temperature fluctuations throughout the year.

*Production methods*: both conventional and organic food production methods were represented in the sample set.

It is also useful to understand the production density of a foodstuff and the number of relevant authentic samples. For example, the Slovenian wine database, established in 1996, contains 25 authentic wine samples covering various geographical regions and varieties. The database is also included in EU Wine Databank. Another example is related to verification of correct labeling of selected fruits and vegetables on Slovenian market including strawberries, cherries, asparagous apples, kaki and garlic. Authentic samples are provided annually by the regional units of the Administration of the Republic of Slovenia for Food Safety, Veterinary, and Plant Protection from producers from various geographical production areas. This research began in 2018 and requires at least 30 authentic samples covering four geographical regions.

Once collected, each sample was carefully labeled with metadata, including information on the geographic origin, date of collection, food type, and any relevant production details. Samples were then prepared for isotopic and elemental analysis according to standardized operational protocols, ensuring consistency in the treatment of all samples.

Although IsoFoodTrack has been designed to cover only a limited area, such as Slovenia, it is crucial to recognize the limitations inherent in regions with sparse data availability on a global scale. Low-data regions may exhibit incomplete coverage, which can limit the robustness of isotopic analysis in those areas. To address this, IsoFoodTrack could adopt the following two strategies to enhance its global applicability: encouraging contributions from other researchers, initiatives that can fill data gaps and build regional datasets and leveraging artificial intelligence (machine learning) to predict isotopic baselines in low-data regions, taking into account appropriate uncertainty metrics.

### 3.2 Isotopic and elemental analysis

The analysis of stable isotope ratios and elemental composition was conducted using precise and well-established techniques. The two main analytical methods used were Isotope Ratio Mass Spectrometry (IRMS) and Inductively Coupled Plasma Mass Spectrometry (ICP-MS) for elemental profiling.

#### 3.2.1 Isotopic analysis

There are only a few exceptions where no sample treatment is needed, such as determining the *δ*^18^O value of water in food and the *δ*^2^H, *δ*^13^C, and *δ*^18^O values of olive oil. However, in most cases, sample treatment is required since it permits the isolation of components that have a stronger geographical fingerprint than the bulk sample and with less interference. For example, samples containing lipids (e.g., meat, fish, milk, and cheese) are usually defatted because fat has a different C and H composition from the other food constituents, and therefore, its variable quantity can affect the overall isotopic signature. In the IsoFoodTrack samples, defatting was performed using a mixture of petroleum ether and diethyl ether (2:1 v/v). Before analysis, all fractions were freeze-dried and stored at room temperature.

Isotope ratios of hydrogen (^1^H/^2^H), carbon (^12^C/^13^C), nitrogen (^14^N/^15^N), oxygen (^16^O/^18^O), and sulfur (^32^S/^34^S) were measured using an isotope ratio mass spectrometer (IRMS) with different preparation systems. The solid and liquid samples are measured by elemental analyser coupled to IRMS (EA/IRMS), ^1^H/^2^H and ^16^O/^18^O in organic matrices with a TC/EA pyrolyser coupled to IRMS, while ^1^H/^2^H and ^16^O/^18^O in water in food samples is determined with MultiFlow Bio equilibration system connected to IRMS.

Validation of a database includes the data within it and its ability to complete the role for which it was designed. All data used to create the database must be validated, i.e., reliable, and all measurements must follow the protocol suggested by Skrzypek et al. (10). The validation tests highlighted in the manuscript were instrumental in assessing its reliability. These tests revealed occasional false positives and misclassifications during bivariate evaluations, particularly in food samples with isotopic compositions near boundary thresholds. For example, foods sourced from regions with similar climatic and environmental conditions exhibited overlapping isotopic signatures. To address these issues, enhanced multivariate analyses were implemented to improve classification accuracy, reducing false-positive rates to below 5% in most categories.

Further, if, upon re-analysis of the samples, data that are consistent with the initial “outlier” data are recorded, further investigation are undertaken to determine the underlying cause. Typically, outliers are due to particular and unique technological or geographical issues, such as a particular microclimate or technological choice. In this case, further investigations are carried out to understand if the outliers belong to another population of data or if they are just “outliers” falling in the percentage of error of the chosen confidence level (for example, 5% for 95% confidence level).

It is strongly recommended that laboratories are accredited to ISO17025 or can demonstrate that they have equivalent quality control systems. This is specifically required if we use the databank to verify the authenticity of commercial samples for food control purposes. According to the norm EN ISO/IEC 17025, the test result of an analytical measurement has to be stated with an estimate of its uncertainty, for example, when uncertainty affects compliance with an authenticity limit. Measurement uncertainty is usually reported in the reference methods (in the case of official and validated methods), or it can be estimated using different methods. Dunn et al. (11) recently published guidance for calculating measurement uncertainty of stable isotope ratio delta values. A further requirement of accreditation is that laboratory must participate in proficiency testing that comply with the ISO/IUPAC/AOAC International Harmonized Protocol for Proficiency Testing of analytical laboratories. In our case this involves participation in the Food Analysis using Isotopic Techniques Proficiency Testing Scheme (FIT PTS), organized by Eurofins Scientific three times per year and includes samples from various food commodities.

#### 3.2.2 Elemental analysis

The elemental composition of each sample was analyzed using Inductively Coupled Plasma Mass Spectrometry (ICP-MS), which is highly sensitive to trace elements. The elemental composition in our samples was performed on triple quadrupole inductively coupled plasma mass spectrometer, QQQ-ICP-MS (Agilent, USA). Prior to analysis, the samples were digested using appropriate chemical methods (e.g., acid digestion) to ensure the accurate quantification of elemental concentrations. Detection limits included in the table were calculated based on three standard deviations of blank measurements. A more detailed description of the optimization of the method for elemental analysis for fruits and vegetables can be found in (12). The elemental data were recorded as concentrations (mg/kg or ppm) for key elements that are relevant to distinguishing different geographical origins and production methods.

Both isotopic and elemental data are stored in the IsoFoodTrack database, alongside the associated sample metadata, to allow for comprehensive comparative analyses. In IsoFoodTrack, isotopic and elemental composition data are represented as single-point measurements.

## 4 Technical aspects

The construction of the database involves three main phases:

- Phase I: the database structure definition. The protocol for data preparation was evolved to minimize the labor involved in populating the database. The database also includes metadata that are important for the further evaluation of the data.
- Phase II: filling the database with data gained during the project evolution.
- Phase III: development of routines/queries for extracting data from the database.

The fundamental requirements of IsoFoodTrack included (i) the underlying database and (ii) the application layer (web application).

### 4.1 Underlying database

The following points were considered in the underlying database:

*Database platform*: the IsoFoodTrack database was implemented using PostgreSQL, an open-source relational database system known for its robustness, support for complex queries, and ability to handle large datasets. The specific technology was chosen for its reliability and synergy with other web technologies. PostgreSQL also enables the storage of semi-structured data when necessary.

*Data import and export*: bulk data import and export were handled using python scripts with PostgreSQL connectors. Data entry was streamlined by importing the isotopic and elemental data from excel file.

Underlying database schema is flexible to mitigate the need for further modification to accommodate growing metadata or analytical results requirements.

A detailed visualization of the database’s structure is provided in the Supplementary Figure 1. This visualization includes an Entity-Relationship (ER) diagram that maps out the database’s tables, the relationships between these tables, and the attributes that define each entity. The diagram identifies primary keys, foreign keys, and various constraints, providing a thorough overview of how data points are linked, referenced, and maintained across the entire IsoFoodTrack ecosystem.

### 4.2 Web application

**Figure 2:**
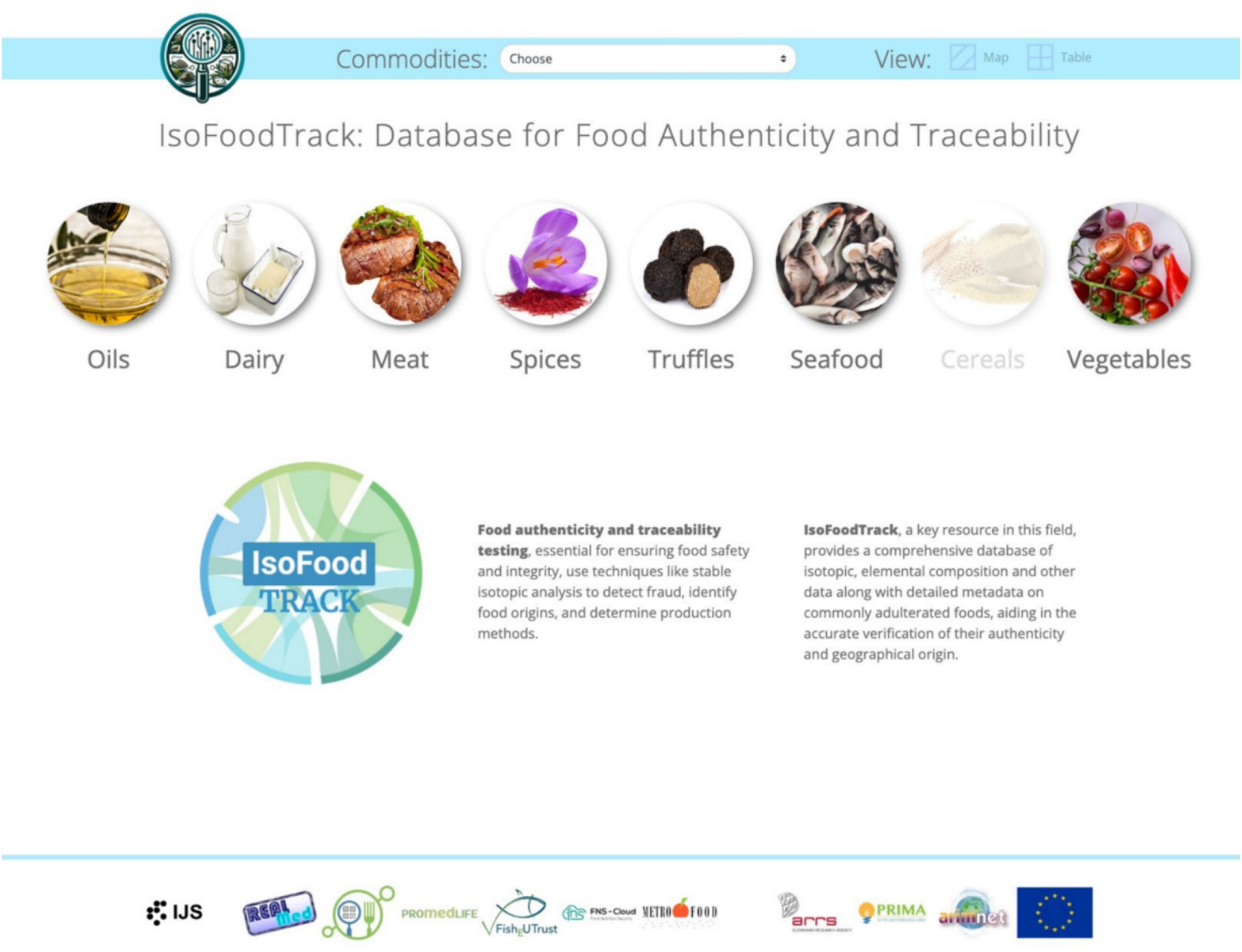
Interface of IsoFoodTrack database.

**Figure 3:**
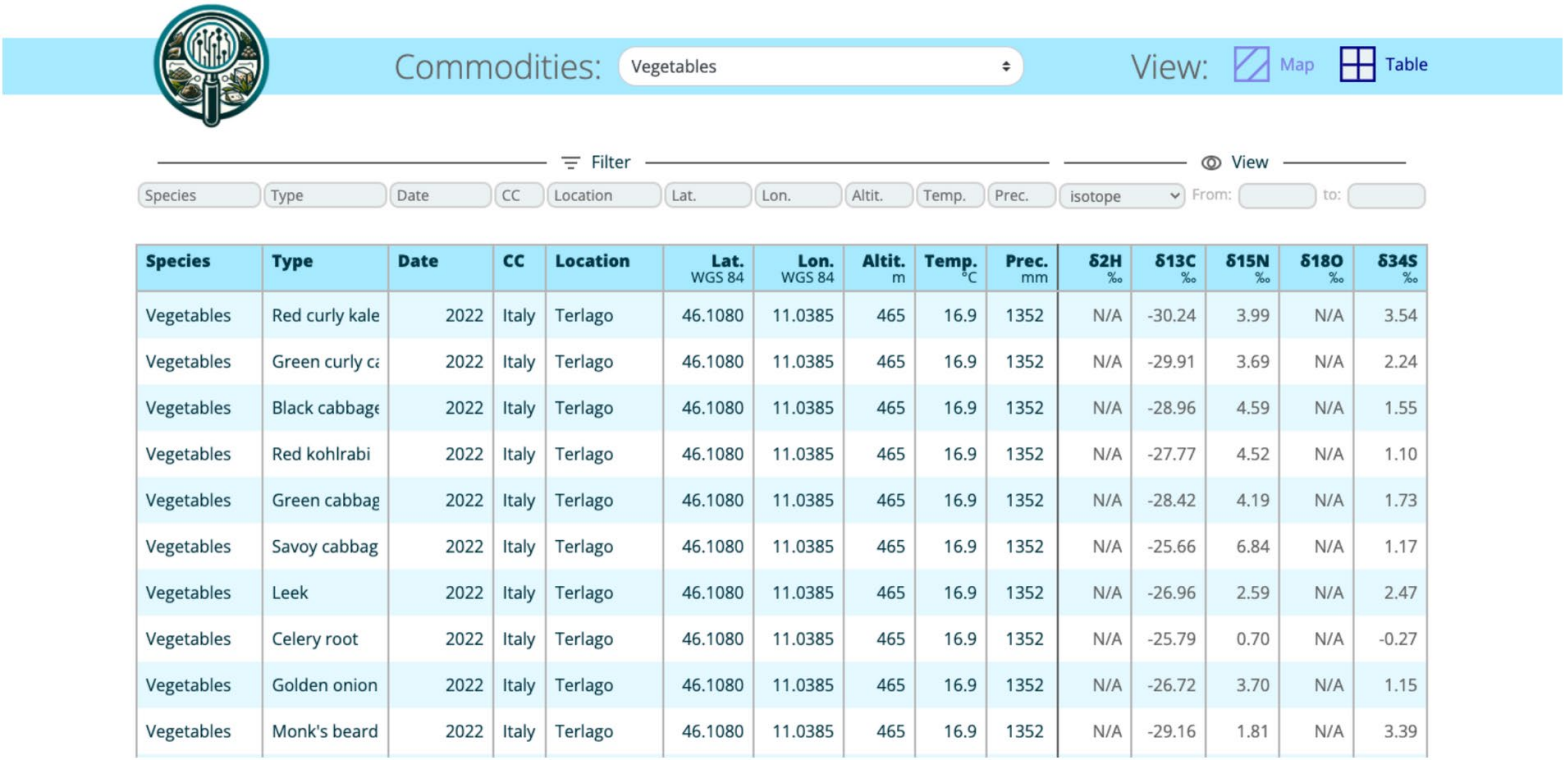
The tabular display of a category.

The web application is a user-friendly interface for interacting and accessing the data from the IsoFoodTrack database. The IsoFoodTrack user interface was built using Django (a Python-based web framework), which allows for integration with the PostgreSQL database. The landing page (Figure 2) is designed to allow users to access the data quickly. All information is organized into categories, and when selecting a category (isotope, elemental composition), a tabular display is presented with the relevant data (Figure 3). The data of elements are grouped into macro, essential, environmental, geological, and toxic categories.

**Figure 4:**
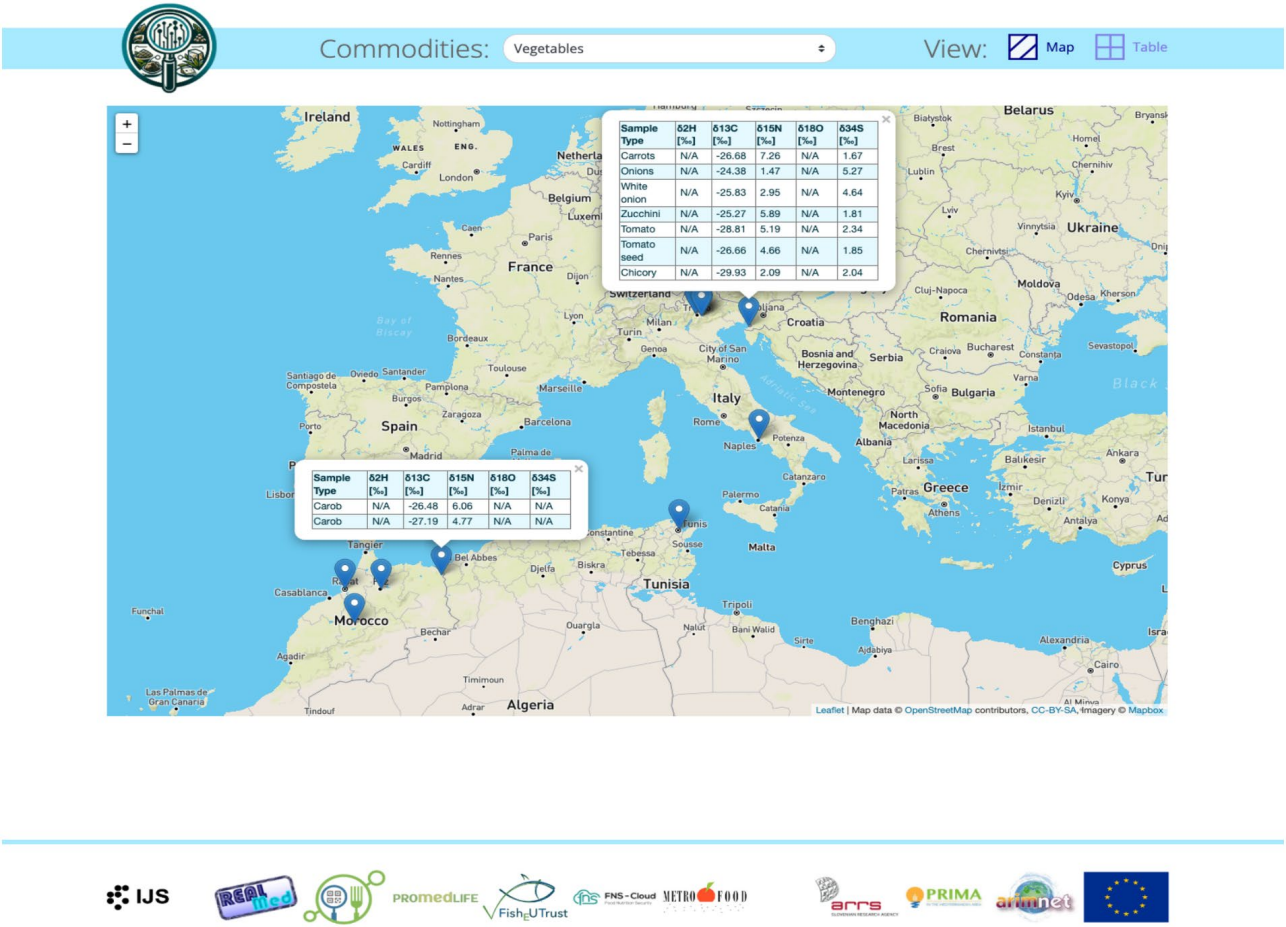
Interactive world map with the data.

The following aspects were considered for the application:

- Security and Auditing (created, updated): The database is accessible to the public only via the web application. Once all data is published, access will be extended to interested users.
- Configuration or metadata and results: Key information, such as column names, data type, units, location data and categorization, are included in the database (Figure 3).
- Review process: Before any new results are added to the database, they are reviewed by the administrator and experts.
- Data Visualization: Django’s ORM (Object-Relational Mapping) was used to query the database and display the results in a user-friendly format. Visualization of data was done using an interactive map with data points representing isotope measurements on different food items from various places around the world (Figure 4). Each data point is clickable and contains relevant information for easier comparison.

### 4.3 Interoperability

Leveraging the data interoperability within the IsoFoodTrack database, an API (Application Programming Interface) can be implemented to allow other systems to interact seamlessly with IsoFoodTrack. This permits external systems or researchers to query the database and retrieve data in standardized formats such as JSON or CSV. The use of standardized data formats and metadata conventions ensures compatibility, facilitating data exchange and cross-referencing with other isotope databases or centralized repositories, like IsoBank. Further, the metadata was selected from the ISO-FOOD ontology (13), which describes isotopic measurements with all of the necessary information required for future analysis. The ontology is linked with standard ontologies, such as Units of Measurement, Food, Nutrient and Bibliographic Ontologies.

**Figure 5:**
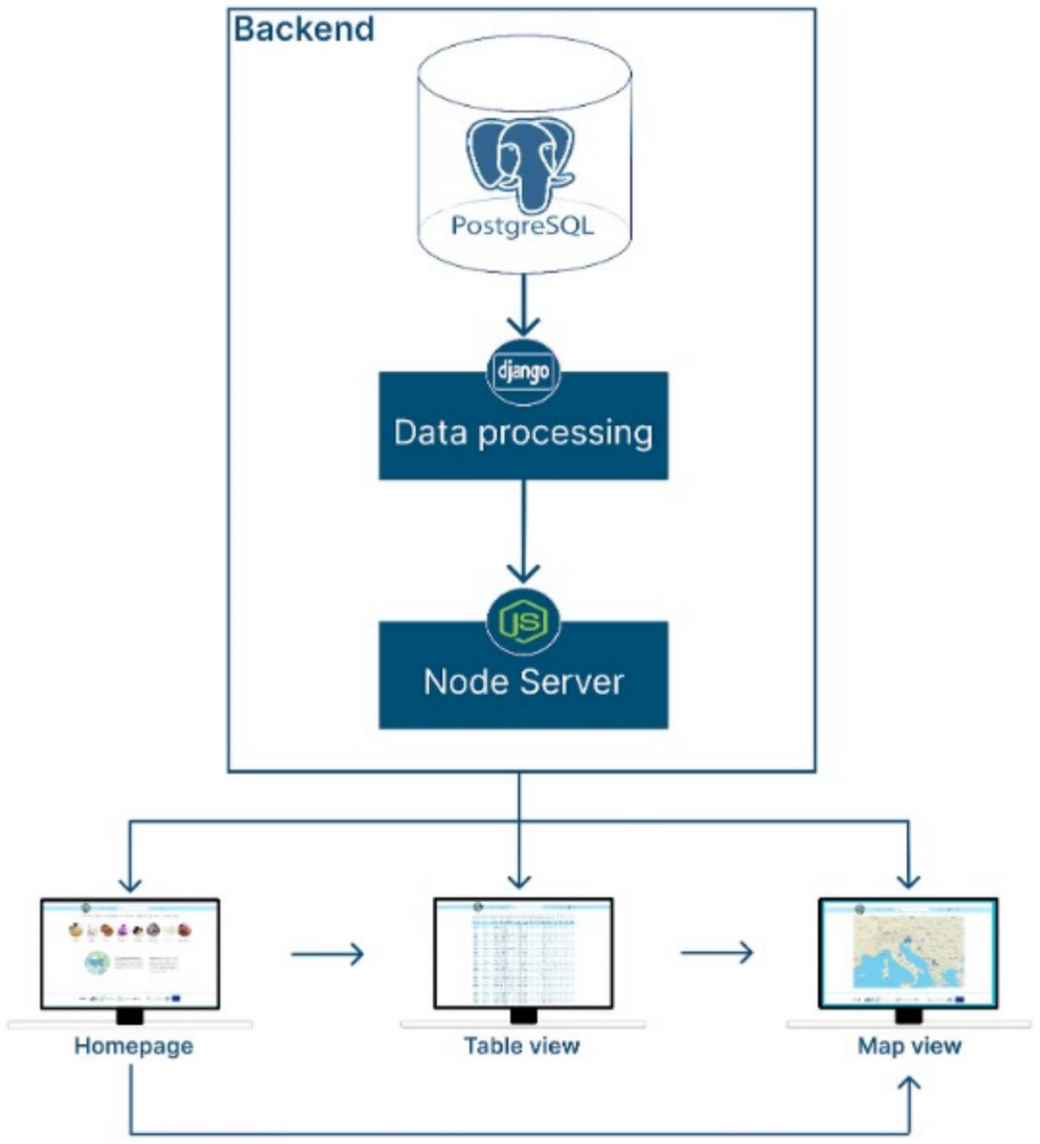
System architecture of the IsoFoodTrack framework.

From a higher-level perspective, the system framework and communication between the web application and its backend processes is presented in Figure 5.

Backend:

- PostgreSQL Database: the central storage of the system, containing the collected and structured data.
- Django (Data Processing): acts as the application layer for data processing and logic implementation, bridging the database and the next layer.
- Node.js Server: serves as the middleware or API server, facilitating communication between the backend and the frontend.

Frontend:

- Bootstrap: framework for IsoFoodTrack views.
- Leafletjs: API that serves map view.
- Jquery: a javascript library.

## 5 Application and validation

Validation of the IsoFoodTrack database was an essential step to ensure its practical application in food authentication and fraud detection. The validation process involved several stages:

### 5.1 Reference dataset validation

In order to reliably determine authenticity, the isotopic data of the test samples must be compared with the databank. The most straightforward and still the most recognized approach is that of univariate data evaluation, based on the arithmetic mean, median, standard deviation and authenticity limits considering the Student’s t-distribution. These metrics enable users to understand the degree of heterogeneity within a given region and enhance the reliability of origin verification. A 95% confidence level is considered appropriate for commercial samples, which are produced in large batches and should have stable isotope values close to the mean values of the authentic materials. The test result can be clear, in terms of true or false, but also suspicious or unlikely, for example, when the reference databank is not robust enough to be considered reliable. The most efficient approach is to create yearly databases, particularly for vegetable and fruit commodities that exhibit significant variability in harvest and production from year to year.

Users should primarily rely on aggregated regional reference values for comparison to account for inherent variability within the dataset. In borderline cases, additional analyses such as examining secondary isotopes or incorporating external metadata (e.g., trace elements or supply chain information) are recommended. For samples classified as “suspicious,” users are advised to conduct further investigations, including re-analysis or consultation with experts.

### 5.2 Statistical analysis

Chemometric methods or multivariate data analysis help separate information from noise, uncover hidden correlations, and visually represent them. There are three main approaches: (1) explorative analysis, (2) classification, and (3) calibration. The choice of method depends on the problem and experimental data (14). For example, principal component analysis (PCA) is commonly used initially for dimensionality reduction, highlighting the most representative features with minimal information loss and generating new variables called principal components (15). However, PCA does not consider group membership, so chemometric methods are used for classification and class modeling when focusing on product origin. Classification, synonymous with discriminant methods, assigns objects to predefined classes using techniques like linear discriminant analysis (LDA), k-nearest neighbors, partial least squares-discriminant analysis (PLS-DA), and artificial neural networks (ANN) (16).

Linear discriminant analysis, one of the simplest classifiers, requires a sufficient ratio (≥3) between samples and variables and struggles with highly collinear data common in chemistry (16). PLS-DA addresses these issues, creating a linear model statistically equivalent to LDA’s solution but overcoming minimum sample-to-variable ratio and collinearity problems (16). Orthogonal partial least squares-discriminant analysis (OPLS-DA), a modification of PLS-DA, enhances interpretability by separating predictive variance from non-predictive variance (17). Model performance is evaluated by explained variation (R2X for PCA and R2Y for OPLS-DA) and predictive ability (Q2), with internal sevenfold cross-validation minimizing overfitting. OPLS-DA prediction performance is measured by sensitivity (true positives) and specificity (true negatives) (18). Discriminant markers are selected by Variable Importance in the Projection (VIP) values, with a threshold of one.

Class modeling, rather than discriminant analysis, is often recommended to confirm a sample’s regional origin due to possible biases in one-class classification problems. Soft independent modeling of class analogy (SIMCA) is a standard method in chemometrics for such tasks (19, 20).

### 5.3 Cross-validation

A cross-validation approach was used to evaluate the robustness of the IsoFoodTrack database. This involved splitting the dataset into training and testing sets, where the training set was used to build a predictive model, and the testing set was used to evaluate the accuracy of the predictions. High predictive accuracy indicated the effectiveness of the database in identifying food fraud and verifying the geographical origin of samples.

### 5.4 Practical applicability

Finally, the practical application of the IsoFoodTrack database was demonstrated by analyzing a set of commercial food samples and comparing their isotopic and elemental profiles against the reference dataset. The results confirmed the ability of the database to detect discrepancies in geographical origin and production claims, thereby validating its utility as a tool for ensuring food authenticity. The Slovenian studies include the use of different chemometric approaches for verifying the geographical region of different commodities. For example, we investigated the possibility of determining the geographical origin of milk and dairy products. Using linear discriminant analysis, discrimination and specification of goat, cow and sheep milk and cheese was possible (21). Moreover, the existing database of authentic Slovenian cow milk was used to verify the correct assignment of regional provenance and declaration of origin. By applying discriminant analysis, the ability to discriminate between geographic regions was only possible when data were organized by year and season as a result of different feeding regimes. Based on the data, a discrimination model was developed to differentiate milk from European milk produced in Slovenia efficiently. Slovenian milk was statistically distinguishable from all other milk, where the most important parameters were *δ*^18^O, Sr., K and *Ca.* Commercial samples labeled as “Slovenian milk” were confirmed and classified as being authentic (22).

Despite the fact that the Slovenian truffles shared some similar characteristics with the samples originating from other countries, differences in the element concentrations suggest that respective truffle species may respond selectively to nutrients from a specific soil type under environmental and soil conditions. Cross-validation resulted in a 77% correct classification rate for determining the geographical origin and a 74% correct classification rate for discriminating between species. The critical parameters for geographical origin discriminations were Sr., Ba, V, Pb, Ni, Cr, Ba/Ca and Sr./Ca ratios, while from stable isotopes *δ*^18^O and *δ*^13^C values are the most important (23).

Discriminant and class-modeling methods have also been applied to assess the geographical classification and authentication of selected fruits and vegetables, including strawberries, cherries, apples, kaki, asparagus and garlic, using stable isotopes of light elements and elemental composition of samples harvested between 2018 and 2020. A good geographical classification of Slovenian and non-Slovenian strawberries was obtained despite different production years using discriminant approaches. Class models generated by data-driven soft independent modeling of class analogy (DD-SIMCA) had high sensitivity (96–97%) and good specificity (81–91%) on a yearly basis, while a more generalized model combining total yearly data gave a lower specificity (63%) (24). Of the 33 commercially available samples (test samples) with declared Slovenian origin, 39% were from outside of Slovenia. The specificity for garlic and asparagus was found to be higher compared to strawberries, indicating that the model for these two commodities is more robust for verifying the correct labeling.

These examples highlight the potential of isotopic and elemental analysis as reliable tools for food origin authentication while demonstrating that some commodities present more significant challenges compared to others.

In addition, the IsoFoodTrack database can be used to support advanced analytics based on statistical and explainable machine-learning approaches, enabling the development of discriminant models to differentiate selected food commodities based on species using elemental and stable isotope data. Machine learning (ML) is a branch of artificial intelligence (AI) that enables systems to learn and improve from experience without being explicitly programmed. The ML component accepts the food’s isotopic composition as input and predicts its geographical origin. Additionally, this approach offers explanations about which specific isotopes serve as indicators for that geographical origin, with the aim of increasing trust in AI-generated suggestions.

The metadata included in the database allow the user to enrich and complement a stable isotope reference database by a more novel approach, i.e., process-based modeling, such as isotopic mapping (isoscapes) (25). Isoscapes can be constructed to make predictive patterns and inform the likelihood of origin based on regional and localized characteristics. The basic concept of isoscapes is reflected in its name, derived from the words “isotope” and “landscape.” Isoscapes visualize the distribution of isotopic ratios (typically of light elements) in space, often using Geographic Information System (GIS) technology to incorporate these ratios into geographic maps. There are two primary methods for producing isoscapes: statistical and process modeling. In statistical modeling, various geostatistical approaches, such as inverse distance weighting and kriging, are used to model the expected isotopic composition of the material in question. These methods typically require extensive databases that densely cover the area of interest. Only a few national-scale isoscapes studies such as wine (26), milk (27), olive oil (28), and rice (29) have been published.

Conversely, process modeling involves obtaining variables with higher spatial density, such as meteorological or geological data, to model the isotopic composition based on the processes affecting isotopic fractionation. For example, food isoscapes are derived from the observation that food produced in a specific area often reflects the local climatic and geological characteristics. The advantage of process-based modeling over statistical modeling is that it requires a much smaller sample database and can be applied to areas with limited sampling. A good example of the spatial variability “GIS modeling Isoscapes” of oxygen and carbon stable isotope composition (*δ*^13^C, *δ*^18^O) of argan oil is also presented by Taous et al. (30). In order to make global, spatially continuous predictions for argan oil stable isotope ratios, the mechanistic models in ArcGIS software (ESRI Corporation ArcGIS 10.5) were implemented. The ordinary point kriging was used to spatially interpolate *δ*^13^C and *δ*^18^O values of argan oils from 25 individual samples collected at five independent regions.

These geospatial models – isoscapes may provide a cost-effective extension to the isotopic dataset approach.

## 6 Database curation and availability

The IsoFoodTrack database (see text footnote 2) curates isotope data from authentic food samples, primarily from Slovenia and other countries, as part of various projects. For instance, in the REALMED project, data includes Moroccan argan oil, Tunisian lamb, and truffles from Italy and Slovenia, while in FishEUTrust, data on sea bream from Malta, Portugal, and Spain is included.

Future upgrades will expand the database to cover additional commodities like honey, nuts, wheat, and cereals. In addition, we also intend to supplement our data with relevant, available open-access data from the literature. Open-access data will also supplement existing datasets such as FoodIntegrity, FNS-Cloud, and METROFOOD-RI, and norms for data-sharing, including embargo periods before public release, will be established. Additionally, it complies with the ISO-FOOD ontology (13), supporting semi-automated integration with data from other relevant sources, and is openly available through the NCBO BioPortal. The nomenclature of the elemental components complies with the CEN Standard of food data.

With new data and new methods for data analysis being integrated into IsoFoodTrack, both the tool and the ontology will enable interoperability with other platforms. The underlying concept of interlinking a dataset with semantic resources serves as an excellent example of open science e-infrastructures that will need to be developed in the future.

## 7 Conclusion

IsoFoodTrack represents one of the initial efforts to compile stable isotope and elemental data for food authenticity and fraud detection. Its robust, scalable, and flexible architecture makes it an invaluable resource for researchers, food control agencies, and global food authenticity initiatives. Here are the key achievements and contributions of IsoFoodTrack:

*Comprehensive data management*: The database integrates isotopic and elemental composition data with rich metadata, encompassing geographical, production, and methodological details.

*Flexibility and scalability*: IsoFoodTrack is designed for continuous expansion, allowing the inclusion of new food commodities, analytical methods, and metadata fields. This ensures the database remains adaptable to ongoing research and technological developments, making it future-proof.

*Interoperability and integration*: The use of standardized formats like JSON and CSV, along with the potential for API integration, enables IsoFoodTrack to exchange data seamlessly with other databases such as future IsoBank. This promotes collaboration and data sharing across global research initiatives.

*User-friendly access and data visualization*: IsoFoodTrack’s web interface, developed using Django, provides an intuitive platform for users to access, query, and interact with the data. Features like interactive maps and tabular displays allow for easy visualization and comparison of data across different geographical locations and samples, enhancing user engagement and analytical capacity.

*Rigorous quality control*: The database implements a thorough validation process to ensure high-quality and reliable data. All samples undergo standardized treatment and analysis, with expert review ensuring that only accurate and validated data is included. This robust approach to quality control enhances the credibility of IsoFoodTrack in scientific research.

*Practical applicability*: Based on the data collected in IsoFoodTrack using statistical, chemometric and machine learning approaches the database has a capability to identify and classify the origin of food commodities. It also supports isoscapes, which visualize isotopic ratios across regions, providing spatially continuous predictions of geographical origins of food products. This novel approach adds depth to isotopic analysis and extends the database’s predictive capabilities, particularly in regions with limited sampling.

*Future enhancements*: Future upgrades include expanding the database to cover more food commodities (e.g., honey, nuts, cereals) and integrating open-access data from literature. These enhancements ensure that IsoFoodTrack will continue to evolve as a critical resource for food authenticity research.

## Data Availability

The raw data supporting the conclusions of this article are available from the corresponding author upon reasonable request.

## Footnotes

^1^Measurements of the stable isotope ratios of light elements are expressed in the *δ*-notation in ‰ according to the equation: *δ*^i^E = (R(^i^E/^j^E)_sample_/R(^i^E/^j^E)_standard_) – 1, where *i* stands for the highest and *j* for the lowest atomic mass number of the element E (C, N, O, S), and R is the isotope ratio between the heavier and the lighter isotope of the element (^2^H/^1^H, ^13^C/^12^C, ^15^N/^14^N, ^18^O/^16^O, ^34^S/^32^S) in the *sample* or *standard*. The *δ*^13^C values are expressed relative to V-PDB (Vienna-Pee Dee Belemnite) standard, *δ*^15^N values relative to AIR, *δ*^34^S values relative to V-CDT (Vienna-Canyon Diablo Troilite) standard and the *δ*^2^H and *δ*^18^O values relative to the VSMOW (Vienna-Standard Mean Ocean Water) standard.

^2^ http://isofoodtrack.ijs.si

## References

[1] Danezis GP Tsagkaris AS Camin F Brusic V Georgiou CA. Food authentication: techniques, trends & emerging approaches. TrAC Trends Anal Chem. (2016) 85:123–32. doi: 10.1016/j.trac.2016.02.026

[2] Kelly S Heaton K Hoogewerff J. Tracing the geographical origin of food: the application of multi-element and multi-isotope analysis. Trends Food Sci Technol. (2005) 16:555–67. doi: 10.1016/j.tifs.2005.08.008

[3] Drivelos SA Georgiou CA. Multi-element and multi-isotope-ratio analysis to determine the geographical origin of foods in the European Union. TrAC Trends Anal Chem. (2012) 40:38–51. doi: 10.1016/j.trac.2012.08.003

[4] Christoph N Hermann A Wachter H. 25 years authentication of wine with stable isotope analysis in the European Union–review and outlook. BIO Web Conf. (2015) 5:02020. doi: 10.1051/bioconf/20150502020

[5] Camin F Bontempo L Perini M Piasentier E. Stable isotope ratio analysis for assessing the authenticity of food of animal origin. Compr Rev Food Sci Food Saf. (2016) 15:868–77. doi: 10.1111/1541-4337.12219, PMID: 33401802

[6] Pauli JN Newsome SD Cook JA Harrod C Steffan SA Baker CJ. Why we need a centralized repository for isotopic data. Proc Natl Acad Sci. (2017) 114:2997–3001. doi: 10.1073/pnas.1701742114, PMID: 28325883 PMC5373358

[7] Meier-Augenstein W Hobson KA Wassenaar LI. Critique: measuring hydrogen stable isotope abundance of proteins to infer origins of wildlife, food and people. Bioanalysis. (2013) 5:751–67. doi: 10.4155/bio.13.36 23534421

[8] Camin F Boner M Bontempo L Fauhl-Hassek C Kelly SD Riedl J. Stable isotope techniques for verifying the declared geographical origin of food in legal cases. Trends Food Sci Technol. (2017) 61:176–87. doi: 10.1016/j.tifs.2016.12.007

[9] Donarski J Camin F Fauhl-Hassek C Posey R Sudnik M. Sampling guidelines for building and curating food authenticity databases. Trends Food Sci Technol. (2019) 90:187–93. doi: 10.1016/j.tifs.2019.02.019

[10] Skrzypek G Allison CE Böhlke JK Bontempo L Brewer P Camin F. Minimum requirements for publishing hydrogen, carbon, nitrogen, oxygen and sulfur stable-isotope delta results (IUPAC technical report). Pure Appl Chem. (2022) 94:1249–55. doi: 10.1515/pac-2021-1108

[11] Dunn PJH Hai L Malinovsky D Goenaga-Infante H. Simple spreadsheet templates for the determination of the measurement uncertainty of stable isotope ratio delta values. Rapid Commun Mass Spectrom. (2015) 29:2184–6. doi: 10.1002/rcm.7376, PMID: 26467231

[12] Potočnik D Hudobivnik MJ Mazej D Ogrinc N. Optimization of the sample preparation method for determination of multi-elemental composition in fruit samples by ICP-MS analysis. Measur Sensors. (2021) 18:100292. doi: 10.1016/j.measen.2021.100292

[13] Eftimov T Ispirova G Potočnik D Ogrinc N Koroušić Seljak B. ISO-FOOD ontology: a formal representation of the knowledge within the domain of isotopes for food science. Food Chem. (2019) 277:382–90. doi: 10.1016/j.foodchem.2018.10.118, PMID: 30502161

[14] Granato D Putnik P Kovačević DB Santos JS Calado V Rocha RS. Trends in Chemometrics: food authentication, microbiology, and effects of processing. Compr Rev Food Sci Food Saf. (2018) 17:663–77. doi: 10.1111/1541-4337.12341, PMID: 33350122

[15] Covaciu FD Moldovan Z Dehelean AA Magdas DA Feher IC Puscas R. Determination of pesticides, elements, and stable isotopes in strawberries. Anal Lett. (2016) 49:2560–72. doi: 10.1080/00032719.2016.1140175

[16] Marini F. Classification methods in Chemometrics. Curr Anal Chem. (2009) 6:72–9. doi: 10.2174/157341110790069592, PMID: 39791164

[17] Rongai D Sabatini N Del Coco L Perri E Del Re P Simone N. ^1^H NMR and multivariate analysis for geographic characterization of commercial extra virgin olive oil: a possible correlation with climate data. Food Secur. (2017) 6:96. doi: 10.3390/foods6110096, PMID: 29112134 PMC5704140

[18] Fiamegos Y Dumitrascu C Papoci S de la Calle MB. Authentication of PDO paprika powder (Pimentón de la Vera) by multivariate analysis of the elemental fingerprint determined by ED-XRF. A feasibility study. Food Control. (2021) 120:107496. doi: 10.1016/j.foodcont.2020.107496, PMID: 33536721 PMC7729827

[19] Oliveri P. Class-modelling in food analytical chemistry: development, sampling, optimization and validation issues–a tutorial. Anal Chim Acta. (2017) 982:9–19. doi: 10.1016/j.aca.2017.05.013, PMID: 28734370

[20] Rodionova OY Titova AV Pomerantsev AL. Discriminant analysis is an inappropriate method of authentication. TrAC Trends Anal Chem. (2016) 78:17–22. doi: 10.1016/j.trac.2016.01.010

[21] Nečemer M Potočnik D Ogrinc N. Discrimination between Slovenian cow, goat and sheep milk and cheese according to geographical origin using a combination of elemental content and stable isotope data. J Food Compos Anal. (2016) 52:16–23. doi: 10.1016/j.jfca.2016.07.002

[22] Potočnik D Nečemer M Perišić I Jagodic M Mazej D Camin F. Geographical verification of Slovenian milk using stable isotope ratio, multi-element and multivariate modelling approaches. Food Chem. (2020) 326:126958. doi: 10.1016/j.foodchem.2020.126958, PMID: 32416418

[23] Hamzić Gregorčič S Strojnik L Potočnik D Vogel-Mikuš K Jagodic M Camin F. Can we discover truffle's true identity? Molecules. (2020) 25:2217. doi: 10.3390/molecules25092217, PMID: 32397327 PMC7248893

[24] Strojnik L Potočnik D Hudobivnik MJ Mazej D Japelj B Škrk N. Geographical identification of strawberries based on stable isotope ratio and multi-elemental analysis coupled with multivariate statistical analysis: a Slovenian case study. Food Chem. (2022) 381:132204. doi: 10.1016/j.foodchem.2022.132204, PMID: 35114619

[25] Bowen GJ. Isoscapes: spatial pattern in isotopic biogeochemistry. Annu Rev Earth Planet Sci. (2010) 38:161–87. doi: 10.1146/annurev-earth-040809-152429

[26] West J Ehlerinwqger J Cerling T. Geography and vintage predicted by a novel GIS model of wine *δ*^18^O. J Agric Food Chem. (2007) 55:7075–83. doi: 10.1021/jf071211r, PMID: 17658829

[27] Ehtesham E Baisden WT Keller ED Hayman AR Van Hale R Frew RD. Correlation between precipitation and geographical location of the δ2H values of the fatty acids in milk and bulk milk powder. Geochim Cosmochim Acta. (2013) 111:105–16. doi: 10.1016/j.gca.2012.10.026

[28] Chiocchini F Portarena S Ciolfi M Brugnoli E Lauteri M. Isoscapes of carbon and oxygen stable isotope compositions in tracing authenticity and geographical origin of Italian extra-virgin olive oils. Food Chem. (2016) 202:291–301. doi: 10.1016/j.foodchem.2016.01.146, PMID: 26920297

[29] Sheng M Zhang W Nie J Li C Zhu A-X Hu H. Predicting isoscapes based on an environmental similarity model for the geographical origin of Chinese rice. Food Chem. (2022) 397:133744. doi: 10.1016/j.foodchem.2022.133744, PMID: 35878556

[30] Taous F Amenzou N Marah H Maia R Maguas C Bahmad L. Stable isotope ratio analysis as a new tool to trace the geographical origin of Argan oils in Morocco. Forensic Chem. (2020) 17:100198. doi: 10.1016/j.forc.2019.100198

