# Supplementary material for: IsoFoodTrack: a comprehensive database and management system based on stable isotope ratio analysis for combating food fraud
Source: Front Nutr. 2025 Feb 19;12:1516521. doi: 10.3389/fnut.2025.1516521 (PMC11879815; doi:10.3389/fnut.2025.1516521)
Supplement: Supplementary file 1 [file Image_1.pdf]

Supplementary Material

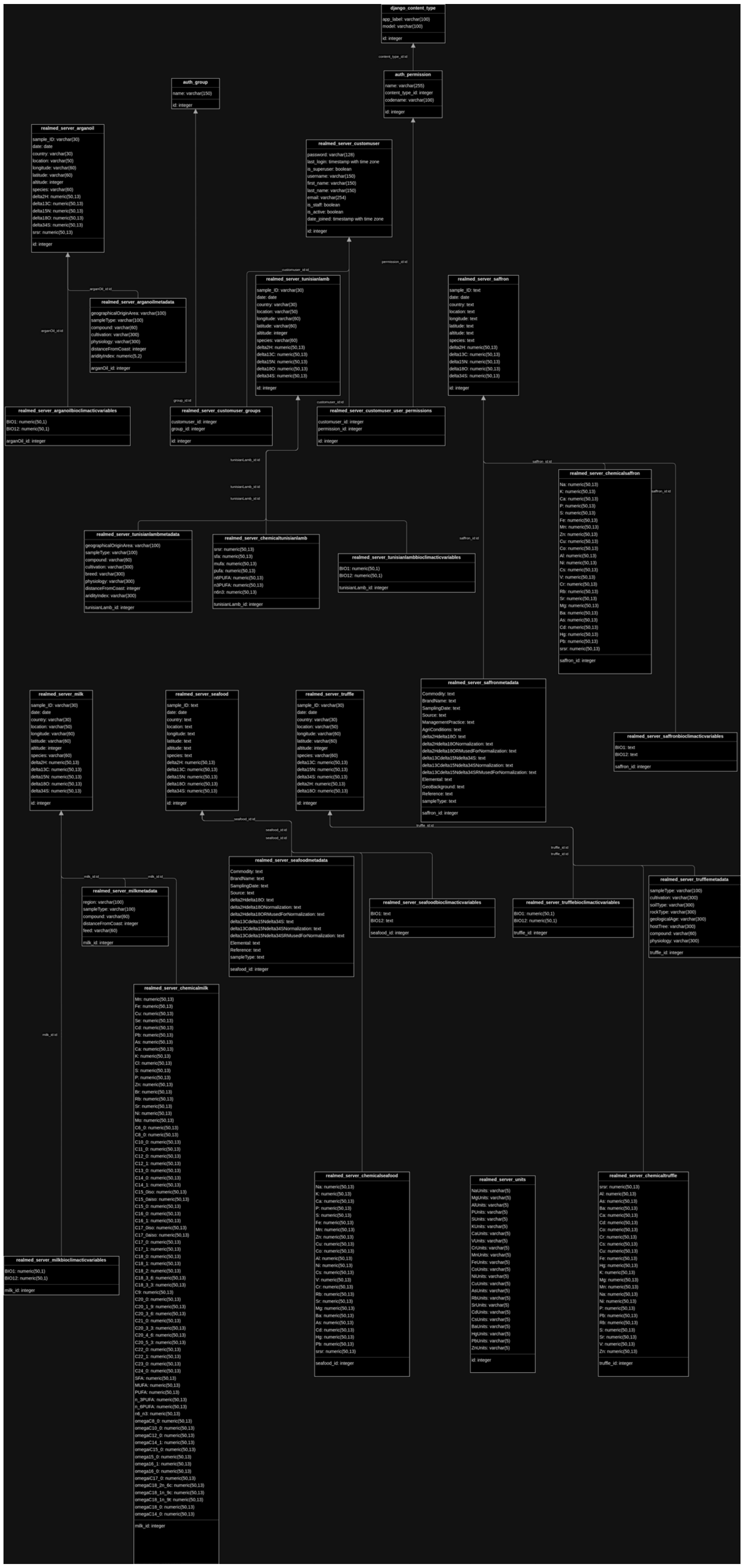

Supplementary Figure 1. A detailed visualization of the database's structure
